# Efficacy of Intravitreal Dexamethasone Implant in Different Patterns of Diabetic Macular Edema

**DOI:** 10.18502/jovr.v15i4.7787

**Published:** 2020-10-25

**Authors:** Claudio Furino, Alfredo Niro, Michele Reibaldi, Francesco Boscia, Giovanni Alessio

**Affiliations:** ^1^Department of Medical Science, Neuroscience and Sense Organs, Eye Clinic, University of Bari, Bari, Italy; ^2^Eye Clinic, Hospital “S. G. MOSCATI”, ASL TA, Taranto, Italy; ^3^Eye Clinic, University of Catania, Catania, Italy; ^4^Department of Surgical, Microsurgical and Medical Sciences, Eye Clinic, University of Sassari, Sassari, Italy

**Keywords:** Dexamethasone Implant, Diabetic Macular Edema, OCT, Ozurdex, Subretinal Detachment

## Abstract

**Purpose:**

Different patterns of diabetic macular edema (DME) suggest different pathogenesis and drug response. We evaluated the outcomes after intravitreal dexamethasone (DEX) implant for DME with or without serous retinal detachment (SRD).

**Methods:**

In this retrospective study, 22 naïve patients (23 eyes) with DME who underwent a single DEX implant were evaluated. Based on the optical coherence tomographic pattern of DME, 12 eyes had a cystoid macular edema pattern (Group 1) and 11 eyes had an SRD pattern (Group 2). The best-corrected visual acuity (BCVA), central retinal thickness (СRТ), central retinal volume (CRV), SRD height (SRDh), and intraocular pressure (IOP) were recorded before and at two and four months after the treatment.

**Results:**

There were no significant differences between the groups regarding demographic, clinical data and outcomes at baseline. In Group 1, the CRT and CRV significantly decreased at two months (*P* = 0.002 and *P* = 0.01, respectively), while the BCVA significantly improved at four months (*P* = 0.03). In Group 2, the CRT and CRV significantly improved (*P* < 0.01 and *P* ≤ 0.01, respectively) during the follow-up period. At four months, both groups showed a recurrence of DME, Group 1 in particular (two-month CRT reduction, –149 ± 127 µm vs four-month CRT reduction, –72 ± 174 µm; *P* = 0.04). The mean reduction in CRV was significantly different at four months (Group 1, –0.49 ± 1.7 mm${}^{3}$ vs Group 2, –1.3 ± 1.3 mm${}^{3}$; *P* = 0.04). In Group 2, the SRDh significantly decreased at two (*P* = 0.01) and four months (*P* = 0.01). Four cases with elevated IOP were managed.

**Conclusion:**

DEX implants were found to be effective in different patterns of DME. The SRD pattern may predict a longer-lasting morphologic efficacy.

##  INTRODUCTION

Diabetic macular edema (DME) is the main cause of visual loss in diabetic patients.^[1]^ Different patterns of nontractional macular edema have been revealed by optical coherence tomography (OCT) including sponge-like swelling, cystoid macular edema (CME), and serous retinal detachment (SRD).^[2,3]^ However, many eyes show more than one macular edema pattern in OCT.^[2,3][4][5][6]^ Different patterns of CME and SRD suggest different mechanisms of pathogenesis,^[7,8,9]^ which lead us to speculate various possible mechanisms of drug action. At present, several pharmacologic approaches are effective for treating DME, including intravitreal anti-vascular endothelial growth factor (VEGF) agents and intravitreal steroid implants. Corticosteroids are effective owing to their anti-inflammatory, antiangiogenic, and blood retinal barrier (BRB)-stabilizing properties.^[10]^ In DME, the sustained-release intravitreal dexamethasone (DEX) implant was observed to be effective, requiring less frequent repeated injections as compared to anti-VEGFs, with common complications such as intraocular pressure (IOP) elevation and cataract formation/progression.^[11]^ In an analysis of data from a Phase II randomized controlled study (NCT00035906), DEX implants were found to display a similar efficacy in different patterns of DME without reference to SRD.^[12]^ The purpose of this study was to analyze the safety and efficacy of a intravitreal DEX implant in the treatment of diabetic CME with and without subretinal fluid.

##  METHODS

In this retrospective study, 23 eyes of 22 diabetic patients with clinically significant DME, as defined by the Early Treatment Diabetic Retinopathy Study (ETDRS),^[13]^ were included. Nontractional DME involving the central macula (1 mm central subfield thickness in the OCT-modified ETDRS grid) with a thickness ≥ 300 μm was considered. Eyes with diffuse spongiform edema, advanced proliferative diabetic retinopathy, other retinal pathologies, or those who underwent previous laser photocoagulation within six months, ocular surgery, or intravitreal injection were excluded. Patient data including age, sex, duration of diabetes, and baseline glycated hemoglobin (HbA1c) levels were recorded. A complete ophthalmologic examination including best-corrected visual acuity (BCVA) measurement, slit-lamp examination, IOP measurement, and fundus examination were performed before as well as two and four months after the treatment. BCVA measured by ETDRS chart was converted into a LogMAR notation for statistical analysis. At each visit, five HD-lines and macular cube (512 × 128) OCT scans were performed using the Cirrus SD-OCT 4000 (Carl Zeiss Meditec, Inc., Dublin, California, USA). The eligible eyes were categorized into two groups according to the OCT pattern of macular edema: Group 1 with CME consisting of intraretinal hyporeflective cystoid spaces and Group 2 with intraretinal edema associated with SRD. The central retinal thickness (CRT, µm) at the foveal site and the central retinal volume (CRV, mm${}^{3}$) for each group were recorded. In Group 2, the maximum SRD height (SRDh, µm) at the fixation point was measured manually using calipers in the “High Definition Image Analysis” mode (HD-OCT Software version 5, Cirrus SD-OCT 4000, Carl Zeiss Meditec, Inc, Dublin, California, USA) and was defined as the average distance between the retinal pigment epithelium (RPE) and the outer neurosensory retinal surface on central vertical and horizontal scans. All OCT scans and measurements were collected by the same observer.

All patients received a single dose (700 µg) of DEX as a sustained-release intravitreal implant (OzurdexⓇ, Allergan, Inc., Irvine, CA, USA). The mean BCVA, CRT, and CRV changes were analyzed at all visits.

The study was approved by the Institutional Review Board (IRB) of an eye clinic in Italy and adhered to the tenets of the Declaration of Helsinki. An IRB approved informed consent was obtained from all patients.

Quantitative data are presented as mean ± standard deviation. Data from the same sample were analyzed using the Wilcoxon test. The differences between the two samples before and after the implantation were assessed using Fisher's test for categorical variables and the Mann–Whitney test for quantitative variables. The sample size was determined considering a confidence level of 95% and a confidence interval of 20. A *p-value* < 0.05 was considered as statistically significant. Statistical analysis was performed using GraphPad Prism, version 5.

##  RESULTS

Twenty-three naïve eyes of 22 patients with type 2 diabetes mellitus were included. The patients included 6 (27.3%) women and 16 (72.7%) men, with a mean age of 62 ± 7.7 years (range, 42–76 years). The mean overall diabetes duration was 6.4 ± 3.4 years, and the HbA1C mean value was 7.5 ± 1% (Table 1). All eyes were phakic with a mild-to-moderate grade cataract. Of the 23 eyes, 12 (52%) had a CME pattern (Group 1) and 11 (48%) had an SRD pattern (Group 2) on OCT analysis.

**Table 1:** Characteristics of patients and their systemic risk factors.


	**Total**	**Group 1**	**Group 2**	**** ***P-*** **value** ${}^{*}$
Female/male (No. of eyes)	6/16 23	4/8 12	2/8 11	*0.43* ${}^{†}$
Mean age (yrs) ± SD	65 ± 10	67 ± 10	64 ± 9	*0.35*
Mean diabetes duration (yrs) ± SD	6.4 ± 3.4	7.2±4.1	4.5 ± 1.6	*0.06*
HbA1C (%)	7.5 ± 1	7.5 ± 0.9	7.4 ± 0.8	*0.78*
SD, standard deviation; HbA1C, glycosylated hemoglobin; DRT, diffuse retinal thickening; CME, cystoid macular edema; SRD, serous retinal detachment ${}^{*}$ *P-*value (Mann–Whitney test); ${}^{†}$ *P*-value (Fisher test) between Groups 1 and 2;* P-*values < 0.05 were considered statistically significant				

**Table 2:** The mean BCVA, CRT, and CRV before and after the treatment at months 2 and 4


	**Total**	**Group 1**	**Group 2**	**** ***P*** -value*
**** ***Baseline*** ****	BCVA (logMAR ± SD) CRT (μm ± SD) CRV (mm${}^{3}$ ± SD)	0.66 ± 0.38 508 ± 141 13.2 ± 1.9	0.66 ± 0.34 478 ± 147 12.8 ± 1.9	0.65 ± 0.43 542 ± 131 13.7 ± 1.8	*0.38 0.38 0.32*
**** ***2${}^{nd}$ month*** ****	BCVA (logMAR ± SD) * P* ${}^{†}$ CRT (μm ± SD) * P* ${}^{†}$ CRV (mm${}^{3}$ ± SD) * P* ${}^{†}$	0.39 ± 0.19 **** *** 0.03 *** **** 337 ± 97 **** ***< 0.0001 *** **** 11.4 ± 1.5 **** ***0.0002*** ****	0.37 ± 0.17 * 0.06* **** ****** **** 329 ± 100 * 0* **** ***.002 *** **** 11.1 ± 1.5 * 0* **** ***.01*** ****	0.42 ± 0.23 * 0.26 * 347 ± 97 **** *** 0.006 *** **** 11.6 ± 1.5 **** *** 0.007*** ****	*0.58 0.77 0.29*
**** ***4${}^{th}$ month*** ****	BCVA (logMAR ± SD) * P* ${}^{†}$ CRT (μm ± SD) * P* ${}^{†}$ CRV (mm${}^{3}$ ± SD) * P* ${}^{†}$	0.36 ± 0.21 * 0* **** ***.02 *** **** 398 ± 119 **** *** 0.001 *** **** 12.4 ± 1.4 **** ***0.001*** ****	0.32 ± 0.19 **** *** 0.03 *** **** 401 ± 133 * 0.15 * 12.3 ± 1.7 * 0.58*	0.39 ± 0.24 * 0.19 * 393 ± 109 **** *** 0.003 *** **** 12.4 ± 1.0 **** *** 0.01*** ****	*0.44 0.97 0.64*
BCVA, best corrected visual acuity; logMAR, logarithm of minimum angle of resolution; SD, standart deviation; CRT, central retinal thickness; CRV, central retinal volume; CME, cystoid macular edema; SRD, serous retinal detachment ${}^{*}$ *P*-values (Mann–Whitney test), comparison between Groups 1 and 2; ${}^{†}$ *P*-value (Wilcoxon test), comparison between baseline and follow-up data (at two and four months) for all patients, and within single groups*; P*-value < 0.05 was considered statistically significant					

**Table 3:** The mean change of BCVA, CRT, and CRV over four months.


	**Total**	**Group 1 (CME)**	**Group 2 (SRD)**	**** ***P*** -value${}^{*}$
BCVA change (0–2 month) (logMAR ± SD) CRT change (0–2 month) (μm ± SD) CRV change (0–2 month) (μm${}^{3}$ ± SD)	–0.31 ± 0.51 **–171 ± 135 **–1.8 ± 1.76	–0.29 ± 0.46 **–149 ± 127 **–1.6 ± 1.7	**–0.23 ± 0.64 **–195 ± 146 **–2.1 ± 1.9	** 0*.71 * *0.34 0.56*
Mean BCVA change (0–4 month) (logMAR ± SD) * P* ${}^{†}$ Mean CRT change (0–4 month) (μm ± SD) * P* ${}^{†}$ Mean CRV change (0–4 month) (μm${}^{3}$ ± SD) * P* ${}^{†}$	–0.30 ± 0.54 * 0.26 *–109 ± 150 ** **** ***0.03 *** **** –0.90 ± 1.56 ** **** ***0.02*** ****	–0.33 ± 0.46 *0.21 *–72 ± 174 ** **** ***0.04 *** **** **–0.49 ± 1.7 * 0.10*	–0.26 ± 0.64 * 0.56 * –149 ± 123 * 0.37 *–1.3 ± 1.3 * 0.10*	*0.64 * * 0.06 * **** ***0.04*** ****
BCVA, best corrected visual acuity; logMAR, logarithm of minimum angle of resolution; SD, standard deviation; CRT, central retinal thickness; CRV, central retinal volume; CME, cystoid macular edema; SRD, serous retinal detachment ${}^{*}$ *P*-value (Mann–Whitney test), comparison between Group 1 and Group 2; ${}^{†}$ *P*-value (Wilcoxon test), comparison within single groups; * P*-values < 0.05 were considered statistically significant				

**Table 4:** Serous Retinal Detachment height changes over follow-up


**Group 2** (11 Eyes)	**Baseline**	**2${}^{nd}$ month**	**4${}^{th}$ month**	**** ***P*** **-value** ${}^{*}$
SRDh (μm ± SD) *P${}^{†}$*SRDh change (μm ± SD)	146 ± 82	72 ± 75 **** *** 0.01 *** **** –74 ± 71	90 ± 61 **** *** 0.01 *** ****–55 ± 59	* 0.38*
SRDh, serous retinal detachment height; SD, standard deviation ${}^{*}$ *P*-value (Wilcoxon test), comparison between SRDh changes at different follow-ups; ${}^{†}$P-value (Wilcoxon test), comparison with baseline; *P*-value < 0.05 was considered statistically significant				

There was no statistically significant difference between the two groups in terms of age, sex, mean diabetes duration, or HbA1C value (Table 1).

The mean overall BCVA, CRT, and CRV improved significantly at two and four months after the DEX implantation (Table 2).

In Group 1, the mean BCVA improved from 0.66 ± 0.34 LogMAR (Snellen equivalent of 20/91) at baseline to 0.37 ± 0.17 LogMAR (Snellen equivalent of 20/47) (*P* = 0.06) at two months and 0.32 ± 0.19 LogMAR (Snellen equivalent of 20/42) at four months (*P* = 0.03). The mean CRT decreased from 478 ± 147 µm at baseline to 329 ± 100 µm at two months (*P* = 0.002) and 405 ± 132 µm at four months (*P* = 0.15). The mean CRV decreased from 12.8 ± 1.9 µm${}^{3}$ at baseline to 11.1 ± 1.5 mm${}^{3}$ at two months (*P* = 0.01) and 12.3 ± 1.7 mm${}^{3}$ at four months (*P* = 0.58; Table 2).

In Group 2, the mean BCVA improved from 0.65 ± 0.43 LogMAR (Snellen equivalent of 20/89) at baseline to 0.42 ± 0.23 LogMAR (Snellen equivalent of 20/53) at two months (*P* = 0.35) and 0.39 ± 0.24 LogMAR (Snellen equivalent of 20/49) at four months (*P* = 0.39). The mean CRT decreased from 542 ± 131µm at baseline to 347 ± 97 µm at two months (*P* = 0.006) and 393 ± 109 µm at four months (*P* = 0.003). The mean CRV decreased from 13.7 ± 1.8 mm${}^{3}$ at baseline to 11.6 ± 1.5 mm${}^{3}$ at two months (*P* = 0.007) and 12.4 ± 1.0 mm${}^{3}$ at four months (*P* = 0.01; Table 2). At baseline and over follow-up, no significant difference was observed in the functional and morphologic outcomes among the groups (*P* > 0.05) (Table 2).

The overall CRT reduction over two months was significantly larger than the reduction observed over four months (*P* = 0.03). In the same way, the overall CRV reduction over two months was significantly larger than the reduction observed over four months (*P* = 0.02). The difference between the mean change in BCVA over the first two months and the overall four months was not statistically significant (*P* = 0.26). At the last follow-up, an improvement of three lines or more was reported in 12 (52%) patients; 7 (58%) in the CME group and five (45%) in the SRD group (*P* = 0.6). The difference between BCVA improvement over the first two and the overall four months within each group and among the groups was not statistically significant (*P* > 0.05). In each group, mainly in Group 1, the mean CRT and CRV reduction after two months of follow-up were larger than those observed four months after the implantation. The mean reduction in CRV was significantly different among the groups four months after the implantation (*P* = 0.04) (Table 3).

In Group 2, the SRDh significantly decreased at months 2 (*P* = 0.01) and 4 (*P* = 0.01) without a significant difference in the mean change at two time points (Table 4). In Group 2, a total resolution of SRD was reported in three (27.3%) eyes and two (18.2%) eyes at two and four months, respectively. Of the three eyes with a resolution of SRD at two months, only one (9%) remained without any SRD at four months. All eyes had a resolution of subretinal detachment without resolution of overlying neuroretinal edema.

Three eyes (13%) had a mild IOP elevation (<25 mmHg), and only one case (3.8%) developed a high IOP elevation (30 mmHg). All cases were medically managed. No other ocular adverse events were reported.

##  DISCUSSION

OCT is the most useful tool for diagnosing and monitoring DME. Based on the OCT features of DME, we focused our study on the therapeutic efficacy of OzurdexⓇ on two different patterns of nontractional DME, namely, the cystoid pattern and the retinal detachment pattern that occur in patients with diabetic retinopathy with a prevalence from 47–55%^[2,4]^ and from 15–31%,^[2,5,6]^ respectively. In our study, both systemic risk factors and morpho-functional data at baseline did not show significant differences among the two OCT-pattern groups, allowing us to evaluate the effectiveness of the drug in two similar populations. Moreover, Vujosevic et al reported that systemic parameters, such as glycemic control and arterial hypertension, did not correlate with the presence of subretinal detachment, thus suggesting that ocular factors might be more important in the development of the features of DME.^[14]^

Intravitreal DEX implants have demonstrated efficacy in the treatment of DME, causing both substantial improvement in BCVA and significant reduction of CRT.^[11]^ In the current study, the mean overall BCVA showed a statistically significant improvement with a larger increase at four months after the DEX implant. An improvement of three lines or more was reported in 52% of patients without differences among the groups. These results are in line with those previously published.^[11,15,16,17,18,19]^ We observed that the mean BCVA of the SRD group remained lower than that of the group with a cystoid pattern over the follow-up period, and that the single-group analysis showed an equal trend of functional improvement without a significant difference in the mean functional recovery between the groups, as previously reported.^[20]^ However, only the eyes with CME showed a significant improvement in visual acuity at four months. Gaucher et al^[21]^ suggested that the presence of subretinal fluid does not seem to be a negative prognostic factor, while other authors have shown that the SRD pattern had a worse prognosis on functional recovery after treatment as compared to the CME pattern^[22,23,24,25,26]^ considering that subretinal fluid could induce photoreceptor degradation which would decrease its metabolism.^[27]^

Overall, the mean CRT and CRV showed a statistically significant reduction at all follow-up visits. The largest reduction in CRT and CRV was observed at two months when DEX reached the highest concentration in the vitreous humor.^[28]^

In both groups, we observed a reduction in the mean CRT and CRV at two months, followed by a moderate increase at four months. The group with a cystoid pattern showed less reduction and a significant recurrence of edema over follow-up, while the eyes with subretinal fluid showed a larger CRT and CRV reduction at two and four months, and a lower recurrence of edema than the eyes with cystoid edema. Deepening the analysis, the SRDh significantly decreased at all follow-up visits, showing the same trend of CRT and CRV. In the SRD group, four patients showed a total resolution of subretinal detachment without resolution of overlying neuroretinal edema at different follow-up visits. The resolution of the subretinal fluid could appear despite persistence or worsening of the neuroretinal swelling and preceding or following its resolution.^[21]^ Horii et al^[9]^ suggested several mechanisms causing different OCT patterns of DME due to BRB breakdown, including the movement of serum proteins, lipids, blood constituents, and small molecules from or to the cystoid spaces. The inner BRB dysfunction, at tight junctions and transendothelial vesicular transport in the capillary endothelial cells, leads to neuroretinal fluid accumulation causing edema.^[7]^ In the cystoid pattern of DME, the occurrence of cysts from degenerating retinal Mϋller cells remains a subject of debate, but their role in the formation of this pattern is certain.^[29]^ An SRD pattern has been postulated to be the result of the movement of fluid from the retina to the subretinal space.^[8]^ The leakage of albumin into the subretinal space brings out fluid, but this does not explain the presence of retinal detachment when the subretinal osmotic pressure equilibrates with the vitreal space.^[30,31]^ Other mechanisms can be involved in favoring subretinal fluid accumulation and might be targeted by steroid therapy. In this regard, external limiting membrane dysfunction can promote the diffusion of proteins into the subretinal space,^[32,33,34,35]^ RPE pump failure found in diabetics and favored by a hypoxic state,^[36,37]^ and reduction of choroidal flow that would induce RPE dysfunction.^[38]^

Corticosteroids have a wide spectrum of anti-inflammatory and anti-edema effects. In particular, these effects include the stabilization of the BRB and increasing the integrity of tight junctions of the endothelial cells of blood vessels, leading to reduction of exudation and downregulation of inflammation.^[39]^ Therefore, our data suggest a greater and more lasting improvement in the morphologic outcomes of CRT and CRV in eyes with an SRD pattern than eyes with CME features after a DEX implant, probably due to a greater stabilization of the outer BRB. Regarding safety, few cases (4/23, 17.4%) had an IOP elevation that could be managed pharmacologically without any surgical approach.

This study has several limitations, including its retrospective, short-term, open-label, uncontrolled nature involving a relatively small number of eyes, which precluded any evaluation of long-term efficacy and need for OzurdexⓇ reinjection. We selected only naïve patients with the aim of evaluating the therapeutic efficacy of DEX implant as the first treatment, even though we are aware that these eyes are more responsive than eyes with DME refractory to anti-VEGF.^[40]^ A comparative analysis of the efficacy of DEX on different OCT patterns of DME between naïve and refractory eyes should be performed.

In conclusion, the anatomical and functional improvements reported in our work could suggest a different therapeutic response of different patterns of DME to slow-release intravitreal DEX implants. Our findings are worthy of investigation in order to develop customized therapies for different tomographic patterns of DME.

##  Financial Support and Sponsorship

None.

##  Conflicts of Interest

There are no conflicts of interest.
